# Vascular Calcification in Chronic Kidney Disease: The Role of Vitamin K- Dependent Matrix Gla Protein

**DOI:** 10.3389/fmed.2020.00154

**Published:** 2020-04-24

**Authors:** Stefanos Roumeliotis, Evangelia Dounousi, Marios Salmas, Theodoros Eleftheriadis, Vassilios Liakopoulos

**Affiliations:** ^1^Department of Internal Medicine, School of Medicine, Aristotle University of Thessaloniki, Thessaloniki, Greece; ^2^Department of Nephrology, Faculty of Medicine, School of Health Sciences, University of Ioannina, Ioannina, Greece; ^3^Department of Anatomy, School of Medicine, National and Kapodistrian University of Athens, Athens, Greece; ^4^Department of Nephrology, University Hospital of Larissa, Larissa, Greece

**Keywords:** chronic kidney disease, dephosphorylated uncarboxylated Matrix Gla protein, end-stage renal disease, Matrix Gla protein, vascular calcification, vitamin K, vitamin K-dependent protein

## Abstract

Arterial calcification is highly prevalent in chronic kidney disease (CKD) patients and is associated with cardiovascular (CV) morbidity and mortality. Patients at early CKD stages are more likely to suffer a fatal CV event than to develop end-stage renal disease and require hemodialysis treatment. The heavy CV burden of these patients cannot be solely explained by traditional calcification risk factors. Moreover, the pathophysiologic mechanisms underlying this association are complex and yet not fully understood. Although vascular calcification was regarded as a passive degenerative process for over a century, this theory changed by recent evidence that pointed toward an active process, where calcification promoters and inhibitors were involved. Matrix Gla Protein (MGP) has been established as a strong inhibitor of calcification both *in vitro* and *in vivo*. Not only it prevents mineralization of the arterial wall, but it is the only factor that can actually reverse it. To become fully active, MGP must undergo carboxylation of specific protein bound glutamate residues, a process fully dependent on the availability of vitamin K. Low vitamin K status leads to inactive, uncarboxylated forms of MGP and has been repeatedly associated with accelerated vascular calcification. Aim of this review is to present the pathophysiologic mechanisms underlying the activation and function of MGP and review the existing, accumulating data regarding the association between vitamin K, MGP and vascular calcification/CV disease in CKD patients.

## Introduction

Cardiovascular (CV) disease (CVD) has an increased incidence and prevalence in patients with chronic kidney disease (CKD) and is directly associated with the high mortality observed in this population. Patients at early stages of CKD are more likely to suffer a fatal CV event than experience deterioration of their renal function to end-stage renal disease (ESRD) and require hemodialysis (HD) (1). The risk for CVD is gradually increased with progression of CKD; reduction of estimated glomerular filtration rate (eGFR) by 10 mL/min confers a 5% increase in the risk for sudden CV death (2), whereas 50% of all deaths in ESRD patients undergoing either HD or peritoneal dialysis (PD) are attributed to CVD (3). This markedly heavy CV burden that accompanies CKD cannot be solely explained by classic risk factors. Novel uremia and dialysis-related factors such as oxidative stress, inflammation and impaired calcium/phosphorus metabolism have been shown to promote vascular calcification (VC) and CVD in ESRD patients (4–8). VC is present even at early CKD stages, is gradually increased with disease progression and is an undisputed independent risk factor for CVD and mortality in uremia. Presence of calcium depositions in the wall of any artery of the human body contributes to a nearly 4-fold risk for CV mortality and a 3.4-fold risk for any CV event (9). Although considered a passive disorder for more than a century, in recent years it became clear that VC is an active, ongoing process involving regulatory proteins and molecules that either promote or inhibit deposition and accumulation of calcium and hydroxyapatite within the vessel wall. Therefore, VC is the result of the imbalance between inhibitors and promoters, where the latter are overwhelming the former. In CKD, VC manifests as intimal or medial calcification, calciphylaxis or mineralization of the heart valves and is characterized by the extremely reduced levels and downregulation of calcification inhibitors (10).

## Matrix Gla Protein: The Powerful Calcification Inhibitor

Although both patients with diabetes and ESRD exhibit a markedly increased prevalence of arterial calcification, 30% of patients with diabetic kidney disease and 17% of ESRD patients undergoing maintenance HD are protected from ectopic mineralization (11, 12), due to the activity of naturally occurring VC inhibitors (10, 12). Matrix Gla Protein (MGP), one of the most powerful inhibitors of VC found in the human body (13). It is a small protein with a molecular weight of 12 kDa containing 84 amino-acids, 5 glutamate (Glu), and 3 serine residues, secreted by cartilage and arterial wall cells. MGP inhibits the process of arterial calcification (14–16), through several pathways (13, 17). MGP is negatively charged and therefore exhibits high affinity for free calcium. Moreover, it can bind directly to circulating calcium molecules and hydroxyapatite crystals that are accumulated within the vessel wall and form inactive complexes. In turn, MGP activates autophagic clearance of these complexes by attracting phagocytes and macrophages (18). MGP can be considered as the usherette that removes free calcium from circulation and leads it to the bones. Besides being a calcium chelator, another molecular pathway through which MGP abrogates VC is the downregulation of bone morphogenetic protein-2 (BMP-2), a well-known VC promoter. BMP-2 triggers transformation of smooth vascular muscle cells to an osteoblastic phenotype and has been identified within the wall of calcified arteries (19). MGP inhibits the binding of BMP-2 to its receptors abrogating thus BMP-2 expression. Non-functional MGP and free activated BMP-2 (that was bound to its receptors) were found in atherosclerotic vessels of aging animal models, whereas molecular inactive complexes of MGP tightly bound to BMP-2 were identified within the arterial wall of healthy arteries (20). The function of MGP was firstly discovered by Luo et al., who developed knockout mice for MGP (MGP^−−/−−^) and found that 6–8 weeks after their birth all of them died from extended aortic calcification (14). Mutations leading to non-functional MGP expression have been repeatedly reported to be associated with Keutel syndrome, a rare condition characterized by excessive, ectopic calcification of soft tissues and cartilage (21). In a cohort of 118 patients with established diabetic nephropathy, followed for 7 years, MGP T-138C (rs rs1800802) polymorphism was found to be a strong, independent predictor of VC and CV mortality (22). Sheng et al., conducted a meta-analysis of 23 studies with 5,773 controls and 5,280 cases and found that MGP G-7A (rs1800801) polymorphism was an independent predictor of both intimal and medial calcification (23). These findings suggest a possible genetic basis in the pathogenesis of VC and molecular activation of MGP.

**Figure 1:**
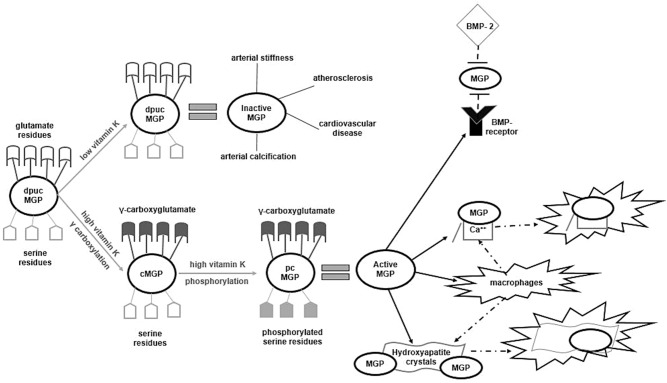
Activation/inactivation processes of Matrix Gla Protein. Dp-ucMGP is the fully inactive form of MGP. If vitamin K is deficient, MGP remains in its inactive form and favors arterial calcification or stiffness, atherosclerosis and subsequent cardiovascular disease. In states of high vitamin K, dp-ucMGP undergoes γ-carboxylation of its glutamate residues and transforms to the cMGP form. In turn, using vitamin K as co-factor, cMGP undergoes phosphorylation of its serine residues and become the fully activated pcMGP. Only in this form, MGP abrogates the connection of BMP-2 to its receptor, tightly binds to free calcium and hydroxyapatite crystals to from inactive complexes and activates autophagic clearance of these complexes by attracting phagocytes and macrophages. MGP, Matrix Gla Protein; dp-ucMGP, dephoshorylated uncarboxylated MGP; cMGP, carboxylated MGP; pcMGP, phosphorylated carboxylated MGP; Ca^++^, calcium anions; BMP, Bone Morphogenetic Protein.

MGP is a member of the vitamin K-dependent proteins (VKDPs) family, a group of 17 human proteins implicated in blood coagulation, VC and bone metabolism (24). All VKDPs have inactive Glu residues and require vitamin K to trigger γ-carboxylation of Glu to γ-carboxyglutamate (Gla). During carboxylation of VKDPs, vitamin K is a co-factor and is further recycled to be re-used for another carboxylation. Transformation of Glu to Gla causes molecular structural and morphologic changes to VKDPs that activates them. Furthermore, after carboxylation of the Gla residues, MGP need to undergo phosphorylation of the serine residues to become biologically active. Phosphorylation is a highly dependent reaction of vitamin K and is thought to be the most critical step in MGP activation. Only after γ-carboxylation and phosphorylation, MGP can gain the ability to bind to calcium, hydroxyapatite and BMP-2 and thus inhibit VC (25) (Figure 1). Therefore, MGP exists in various forms in the circulation, depending on which transformations it underwent: the fully active carboxylated and phosphorylated MGP, the fully inactive dephosphorylated, uncarboxylated form (dp-ucMGP), the partially inactive dephosphorylated carboxylated MGP (dp-cMGP) and the partially inactive uncarboxylated but phosphorylated form (ucMGP or pucMGP). The scientific team from Maastricht were the first to develop specific antibodies that allowed the quantification of total uncarboxylated MGP. Roijers et al. (26), collected 12 tissues of human coronary arteries during autopsies and assessed the microcalcification composition status of the samples with a proton microscope. All atherosclerotic lesions were classified in 4 subtypes, according to the severity of microcalcification: type I were pre-atheromatic lesions and type II to IV exhibited a gradual extensive calcification of the intimal layer. Although no ucMGP was detected in type I lesions, staining for ucMGP exhibited increased intensity in types II to IV. In contrast, staining for total carboxylated MGP and BMP-2 was weak in type I and was considerably increased in type IV lesions. Both carboxylated MGP and BMP-2 were correlated with the degree of microcalcification. The authors suggested that MGP acts as a local calcification inhibitor by binding directly to BMP-2; moreover, delayed or impaired carboxylation of MGP was accompanied by early subclinical microcalcifications in the coronary arteries (26, 27). Since MGP needs vitamin K to become biologically active, Schurgers et al. (17), showed that in animal models, undercarboxylation of MGP -due to 6 weeks treatment with the vitamin K antagonist warfarin- was associated with accelerated arterial calcification. To explore whether existing VC could be reversed by vitamin K intake, all rats were divided to receive either warfarin or vitamin K in low or high dosage for another additional 8 weeks. Compared to rats supplemented with vitamin K, the warfarin group showed accelerated VC, high atherogenic status and significantly higher levels of circulating ucMGP, whereas supplementation with high dosages of vitamin K caused a 37% regression of VC status. The authors concluded that vitamin K -mediated carboxylation of MGP is an essential step in the activation of this powerful natural inhibitor of VC. This was the first *in vivo* study showing that supplementation with vitamin K could possibly stop and even reverse arterial calcification (17). However, despite the initial favorable results in both *in vitro and in vivo* studies, population-based studies failed to show clear-cut results regarding the association of ucMGP with VC and CVD (28–31). This could be attributed to the fact that the quantification method that measured ucMGP was not sensitive for MGP phosphorylation status, since it assessed both dp-ucMGP and pucMGP. It became evident that compared to non-phosphorylated, phosphorylated forms of MGP (regardless carboxylation status) exhibited a significantly higher affinity for binding free calcium, hydroxyapatite crystals and BMP-2 and therefore had a different impact on the development of VC. Moreover, it became clear that phosphorylation of the serine residues was a crucial step of MGP activation (32, 33). The development of specific-sandwich antibodies that allowed quantification of dp-ucMGP separately from other MGP forms showed that compared to ucMGP, circulating d-pucMGP is a more reliable indicator of vitamin K status, a stronger marker of arterial calcification and a better predictor of CVD (13, 33, 34).

In the general population, dp-ucMGP has been repeatedly and strongly correlated with various markers of arterial calcification (35–38), arterial stiffness (39) and CVD (40–42). Similar results were reported in cohorts characterized by high atherogenic status, such as patients with heart failure and CVD (30, 43–45). Since CKD is a state of accelerated calcification of both intimal and media layer as well as soft tissues, several investigators explored the association between dp-ucMGP and VC /CVD in these patients. The Maastricht group was the first to conduct a cross-sectional, prospective study in 107 uremic patients stratified in various stages of CKD (2–5) and found that circulating dp-ucMGP was strongly associated with aortic calcification score, deterioration of renal function and all-cause mortality (32). In a cohort of 67 patients with diabetic CKD in stages 2-5, d-pucMGP was gradually increased with disease progression to ESRD and strongly predicted all-cause and CV mortality (22). Similarly, in CKD populations, several investigators reported a tight association between circulating dp-ucMGP and various VC markers and renal function (29, 46, 47). Likewise, in HD patients, there is a growing body of evidence showing a strong, independent association between dp-ucMGP levels and CV mortality and morbidity (48–51). Since increased dp-ucMGP reflects poor vitamin K status and has been shown to predict CVD and mortality, it has been hypothesized that supplementation with vitamin K might ameliorate VC, through activation of MGP. However, these studies had observational design, small sample sizes and used various surrogate markers of VC as endpoints and not hard CV outcomes. Moreover, they did not assess directly vitamin K status, but hypothesized that dp-ucMGP reflected vitamin K deficiency.

## Vitamin K: The Essential Co-Factor of MGP

Vitamin K refers to a family of fat-soluble vitamins, involved in blood clotting, bone metabolism and regulation of free calcium. Vitamin K consists of two natural vitamers: K1 and K2. K2, in turn includes various chemical subtypes, the menaquinones (MK) (52). Compared to all other forms and subtypes, MK-7 exhibits the highest efficacy in humans due to its better bioavailability and longer half-life (53). The clinical importance of vitamin K2 lies to their role as crucial co-factors for the complete synthesis of VKDPs. Vitamin K-mediated conversion of Glu residues to Gla, allows VKDPs -such as MGP- to became biologically active and play pivotal role in bone metabolism, blood coagulation and vascular health.

There is a growing body of evidence from population-based studies suggesting a tight association between vitamin K intake and CV mortality and morbidity. The Rotterdam study enrolled 4,807 subjects with no history of myocardial infarction at baseline. After 10 years of follow-up, the risk for aortic calcification, all-cause mortality and incident CVD was significantly, independently and negatively correlated with daily dietary intake of K2. However, K1 consumption failed to show any association with any of the end-points (54). In agreement with these results, data from the Third National Health and Nutrition Examination Survey (NHANES III) and Prevention of Renal and Vascular End-Stage Disease (PREVEND) studies showed that vitamin K deficiency was a strong, independent predictor of CVD, mortality and incidence of CKD (55, 56). Chen et al., conducted a meta-analysis of 21 studies including 222,592 subjects and found that both daily dietary consumption of K1 and K2 were associated with aortic calcification (57). Several studies reported that CKD patients present with a significant -often underdiagnosed- deficiency of vitamin K, which is gradually increased along with deterioration of renal function and is more pronounced in dialysis patients. In this population, poor vitamin K2 status is reflected by increased circulating dp-ucMGP levels and predicts accelerated arterial calcification, CV morbidity and mortality (13, 22, 32, 58–60).

Exogenous supplementation with vitamin K exhibited promising results in animal models. Four weeks of K1 and K2 treatment in rats with renal failure and enhanced arterial calcification resulted in significant decrease of aortic calcium (61). Animals treated with warfarin exhibited accelerated aortic calcification due to a 5-fold downregulation of circulating MGP. Moreover, supplementation with K1 and K2 resulted in a significant 50% decrease in aortic calcium and upregulation of MGP levels (17). Similarly, in animals with warfarin-induced VC, supplementation with K2 -but not K1- resulted in significant reduction of arterial mineralization; while, in the thoracic aorta, concentration of K2 was found to be three times higher than K1 (62). In another study, uremic rats with chemically-induced VC were randomized to either low or high MK-7 intake for 3 months. High MK-7 supplementation resulted in a significant reverse of CV mineralization, compared to low intake, probably through a 10-fold upregulation of MGP expression in the aorta (63). Therefore, data from experimental animal models suggested that supplementation with vitamin K (especially K2/MK-7) might abrogate development of VC.

After the promising results that were reported in animal models, several investigators aimed to explore the possible effect of vitamin K (especially MK-7) on the inactive form of MGP, in human interventional studies. Randomized, placebo-controlled studies in the general population reported that although dp-ucMGP levels of the placebo group remained unchanged or even increased, the groups that were supplemented with MK-7 exhibited a significant 32–36% decrease in dp-ucMGP concentrations (36, 64). In a dose-response, randomized, double-blind, placebo-controlled study, 42 healthy subjects were allocated to receive placebo or MK-7 at a daily dose of 10, 20, 45, 90, 180, or 360 μg for 3 months. After the follow-up period, the placebo group exhibited a 16.8% increase in circulating dp-ucMGP levels, whereas even the group that received the smallest amount of MK-7 (10 μg) presented a 12.1% decrease. Moreover, compared to low intake, subjects that were supplemented with high daily dose of MK-7 (180 or 360 μg) had a 39.7 and 56% decrease of dp-ucMGP levels, respectively (65). The authors concluded that high MK-7 intake could improve carboxylation status of MGP and therefore might ameliorate development of VC. Similar results were reported in CKD patients. Kurnatowska et al. (66), randomized 42 CKD patients to daily intake of 90 μg/day intake of MK-7 combined with 10 μg of vitamin D or D alone for 9 months. After the treatment period, the group that received only vitamin D exhibited an increase in dp-ucMGP and a decrease in MGP levels, whereas the combined therapy group presented a 19% decrease of dp-ucMGP and an increase in circulating MGP levels (66). In another study, 53 HD patients were randomized to daily supplementation of 45, 135, or 360 μg of MK-7 for 6 weeks. The authors found that MK-7 supplementation had a dose- and time-dependent reduction in dp-ucMGP levels (60). In a pre-post intervention study including 50 HD patients, Aoun et al., found that supplementation with high dose of MK-7 (360 μg/day for 4 weeks) resulted in a significant 86% reduction of dp-ucMGP levels (67).

Following these results, the scientific research was focused on the effect of vitamin K supplementation on CV morbidity and mortality, in high atherogenic populations, such as uremic patients. In the study by Kurnatowska et al., it was shown that daily intake of 90 μg of MK-7 resulted in a slower progression of VC in a cohort of 42 CKD patients (66). Large daily dose of 360 μg of MK-7 for 2 months was accompanied by a 14.2% reduction of arterial stiffness in a cohort of 60 kidney transplant recipients (68). Lees et al. (69), conducted a meta-analysis including 13 controlled clinical studies and 14 longitudinal trials in 12,888 patients and found that compared to control, vitamin K supplementation was associated with a 9.1% reduction in VC status, accompanied by a 44.7% decrease in dp-ucMGP levels. However, vitamin K caused a non-significant improvement in arterial stiffness. Moreover, in longitudinal studies, dp-ucMGP was found to be an independent predictor of CV morbidity and mortality. The authors suggested that larger, controlled trials should test the effect of vitamin K intake -through carboxylation of dp-ucMGP- on VC and CVD in uremic patients (69). The design and results of the intervention studies that assessed the effects of vitamin K supplementation on MGP forms and various endpoints are summarized in Table 1. These studies have several limitations: none of them is randomized, placebo controlled trial, the sample sizes are quite small, the doses of MK-7 intake differs among studies and finally no hard CV outcomes are assessed, but only surrogate markers of VC.

**Table 1:** Interventional clinical trials regarding the effects of vitamin K2 (MK-7) supplementation on circulating forms of MGP and arterial calcification and stiffness.

**Study design, population, year, reference**	**Intervention**	**Study duration**	**Result**
Single-arm, clinical, 17 HD patients, 2011, (51)	135 μg/day MK-7	6 weeks	27% ↓ of dp-ucMGP No effect on dpcMGP
Randomized, non-placebo controlled, clinical, 53 HD patients, 2012, (60)	45, 135, or 360 μg/day MK-7 in three groups, respectively	6 weeks	45 μg group: 17.9% ↓ of dp-ucMGP 135 μg group: 36.7% ↓ of dp-ucMGP 360 μg group: 61.1% ↓ of dp-ucMGP
Prospective, randomized, single-blinded, clinical, 200 HD patients, 2014, (28)	360, 720, or 1080 μg/thrice weekly MK-7 in three groups, respectively	8 weeks	360 μg group: 17% ↓ of dp-ucMGP 720 μg group: 33% ↓ of dp-ucMGP 1080, μg group: 46% ↓ of dp-ucMGP
Randomized, double-blind, clinical, 40 non-dialyzed CKD patients stage 3–5, 2015, (66)	10 μg/day vitamin D+ 90 μg/day MK-7 or 10 μg/day vitamin D	9 weeks	D group: ↑ of dp-ucMGP, ↓ of MGP MK-7+D group: ↓ of dp-ucMGP, ↑ of MGP, slower progression of vascular calcification compared to the vitamin D group
Pre-post, clinical, 50 HD patients, 2017, (67)	360 μg/day MK-7	4 weeks	↓ 86% of dp-ucMGP
Single-arm, single-center, clinical, 60 kidney transplant recipients, 2017, (68)	360 μg/day MK-7	8 weeks	55.1% ↓ of dp-ucMGP 14.2% ↓ of arterial stiffness 40% ↓ of the prevalence of subclinical vitamin K deficiency

*MK-7, menaquinone 7; MGP, Matrix Gla Protein; Dp-ucMGP, dephosphorylated, uncarboxylated MGP; D-pcMGP, dephosphorylated, carboxylated MGP; HD, hemodialysis; CKD, chronic kidney disease*.

## The Main Areas of Debate and Research Questions

There are still several areas of debate regarding the interplay between MGP and vitamin K in CKD patients. Firstly, the ideal method of vitamin K status assessment remains under investigation. Direct measurement of vitamin K1 in serum or plasma does not reflect K2 levels and circulating K2 might not reflect the availability of the vitamin for VKDPs' activation. A collective indicator of all uncarboxylated VKDPs, the Protein Induced by Vitamin K Absence or Antagonism (PIVKA) and circulating dp-ucMGP are currently considered the most reliable markers of vitamin K availability and status (70). However, very few studies have assessed the association between these markers and CV outcomes in CKD populations. Secondly, the interactions of vitamin K with other medications are yet to be elucidated. In HD and renal transplant recipients, *in vitro* and clinical data suggest that phosphate binders might enhance vitamin K bioavailability, through undesired binding of K2. Thirdly, it is not yet determined whether dp-ucMGP is a marker of accelerated arterial calcification (32), arterial stiffness (39, 45) or both. Finally, the side-effects and the toxicity of vitamin K supplementation need to be further clarified. To date, it has been shown that even in large doses, no toxicity or increased risk of thrombosis with vitamin K1 or K2 supplementation has been documented. However, the results of the current on-going interventional, clinical trials will shed some light in the safety and side-effects of vitamin K supplementation in large cohorts of CKD and HD patients.

Currently, there are several on-going, multi-center, randomized, placebo-controlled studies investigating whether supplementation with K1 or K2 can slow progression of VC and protect from CVD in CKD or HD patients, such as: the KING trial (68), the iPACK-HD Study (71), the VitaVasK (72), the VitK-CUA-NCT02278692 (73), the Vita-K 'n' CKD Study-NCT03311321 (74), the TReVasc-HDK-NCT02870829 (75), and the RenaKvit study-NCT02976246 (76).

## Main Points, Take-Home Messages and Future Directions

MGP, one of the most natural powerful inhibitors of VC needs vitamin K to become active. Poor vitamin K status has been repeatedly established in CKD and dialysis patients. Currently, there are no recommendations regarding vitamin K supplementation in CKD and HD patients. However, there is a growing body of evidence suggesting that vitamin K supplementation might abrogate VC and thus development of CVD through activation of MGP. In all interventional studies so far, no toxicity or serious side-effects of vitamin K intake have been reported, therefore it is considered a safe therapy with potentially important clinical impact. Several areas of research in this topic remain unanswered: should CKD and HD patients be monitored for vitamin K deficiency? If so with which method? Should they receive vitamin K supplements? If so vitamin K1 or K2 and in what dose? Several randomized studies are currently investigating this possible anti-atherogenic effect of vitamin K intake. The results of these studies might shed some light in this area and recommendations for daily intake of vitamin K in CKD and HD patients might be re-considered.

## Author Contributions

VL conceived the idea, prepared the final version, and finalized the manuscript. SR searched the literature and prepared first part of the first draft. ED prepared the second part of the first draft. MS reviewed the first draft and prepared the revised version. TE reviewed the literature, critically reviewed the final version, and approved the final manuscript. All authors have substantially contributed to the preparation of the manuscript and are in agreement with the study.

## Conflict of Interest

The authors declare that the research was conducted in the absence of any commercial or financial relationships that could be construed as a potential conflict of interest.
